# Latent profiles of post-traumatic growth in patients with recent hysterectomy: psychosocial predictors and stigma-associated outcomes

**DOI:** 10.3389/fpsyt.2025.1552946

**Published:** 2025-07-28

**Authors:** Yuqing Sun, Shirong Wu, Qunfeng Zou, Xiuzhen Fu, Silu Liu, Gongna Chen, Xiaojie Zheng, Dingrong Qiu

**Affiliations:** ^1^ The Second Clinical School of Medicine, Guangzhou University of Chinese Medicine, Guangzhou, China; ^2^ Department of Nursing, The Second Affiliated Hospital of Guangzhou University of Chinese Medicine, Guangzhou, China; ^3^ Department of Sports Medicine, The Second Affiliated Hospital of Guangzhou University of Chinese Medicine(Er Sha Island), Guangzhou, China

**Keywords:** hysterectomy, latent class analysis, patients, post traumatic growth, psychological, social stigma

## Abstract

**Background:**

Studies have found that post-traumatic growth exists in patients with recent hysterectomy. However, previous studies have overlooked heterogeneity within groups. There is a lack of research on whether there are different categories of post-traumatic growth levels in patients with recent hysterectomy. Therefore, this study explores different categories of post-traumatic growth and their influencing factors in patients with recent hysterectomy based on latent profile analysis. It also analyses the relationship between stigma and different profiles of post-traumatic growth in patients.

**Methods:**

This study, which used convenience sampling to select patients who underwent hysterectomy at the gynaecology department of a tertiary hospital in Guangzhou, Guangdong Province, was conducted from February to September 2024. The study included 210 patients aged 18 years or over who had undergone a hysterectomy and had agreed to participate in the questionnaire survey. A total of 210 questionnaires were distributed and 202 valid responses were received. The study used the following scales: a general information questionnaire (covering patients’ sociodemographic and clinical characteristics); post-traumatic growth inventory-Chinese version (C-PTGI); and social impact scale (SIS). The C-PTGI primarily measures the level of posttraumatic growth in patients with recent hysterectomy; higher total scores indicate greater posttraumatic growth. The SIS assesses the level of stigma experienced by patients with recent hysterectomy, with higher scores indicating a greater degree of stigma. The data were analysed using SPSS 25.0 and Mplus 8.3.

**Results:**

Post-traumatic growth in patients with recent hysterectomy consisted of 3 types: negative growth group (18%), low-transformation-moderate growth group (53%), and positive growth group (29%). Significant differences in post-traumatic growth and scores on each dimension were found in different subgroups (*P* < 0.05). Patients with recent hysterectomy aged 40–59 were more likely to be classified as belonging to the positive growth group. Those with high scores on the internalized shame and social isolation dimensions were more likely to belong to the negative growth group. Compared with the positive growth group, educational attainment at elementary or junior high school level was a predictor of belonging to the low-transformation-moderate growth group.

**Conclusion:**

Heterogeneity exists in the post-traumatic growth level of patients with recent hysterectomy. Younger patients with higher levels of education and lower scores on the SIS tend to experience better post-traumatic growth after hysterectomy. Healthcare professionals should adopt more flexible and targeted interventions to help patients enhance their post-traumatic growth levels under the premise of correctly identifying the characteristics of different potential categories. This can better improve the mental health status of patients, promote their postoperative recovery, and enhance their quality of life and satisfaction.

## 1 Introduction

Hysterectomy is a primary and preferred treatment option for gynaecological diseases (1), such as benign conditions like uterine fibroids, endometriosis and uterine prolapse, as well as malignant conditions like cervical and ovarian cancer. Epidemiological surveys show that approximately 600,000 women undergo hysterectomies each year in the United States alone (2). Within the European Union, more than 400,000 patients undergo hysterectomies each year due to disease (3). In China, the number of total hysterectomies performed each year is as high as 2.8 million (4). While surgery can alleviate the pain caused by the disease, it is important to acknowledge the numerous effects that hysterectomy can have on patients.

Hysterectomy can cause damage to the pelvic floor structure, decreased ovarian function, and impaired sexual function, increasing the risk of pelvic floor dysfunction, urinary dysfunction, and sexual dysfunction, as well as premature or exacerbated perimenopausal symptoms (5–7). In addition, hysterectomy surgery can lead to decreased oestrogen and progesterone secretion, enhancing patients’ perception of negative emotions (8, 9). Patients may also experience pressure from various sources, such as family and society, which can make them more susceptible to psychological issues. Previous studies have shown that women who undergo hysterectomy are at higher risk of long-term mental health problems such as anxiety and depression than those who do not undergo hysterectomy (10). The majority of patients with recent hysterectomy are in the perimenopausal period, and their symptoms may also affect their psychological state (11). A longitudinal study found that, compared to women who did not undergo a total hysterectomy, those who underwent the procedure while preserving their ovaries had a 20% increased risk of depression. Meanwhile, those who underwent the procedure without preserving their ovaries had a 44% increased risk (12). Casarin et al. (13) reported anxiety prevalence rates of 47%, 29% and 38% at preoperative, postoperative hospitalisation and 3 months postoperatively, respectively, while depression prevalence rates were 11%, 14% and 28%, respectively. A study by Wang Yue et al. (14) showed that anxiety rates among patients who underwent a total hysterectomy could be as high as 79.9%, with depression rates reaching 52.2%. The above research indicates that, as a treatment method that causes organ damage, hysterectomy, regardless of the route or method used, will affect the psychological and physiological health of patients. Therefore, the practical urgency of improving the mental health of patients with recent hysterectomy is remarkable. However, research on the mental health of patients who have undergone hysterectomies remains limited at present.

As the field of positive psychology continues to develop, researchers have come to realize that mental health research should not only focus on how to reduce mental illness, but also on individuals’ positive psychological characteristics (15). Post-traumatic growth (PTG) is typically defined as the positive changes that occur in individuals following traumatic experiences (16). According to the emotional-cognitive processing model of PTG, when individuals experience trauma, they make cognitive evaluations and adopt various emotional responses and coping strategies. These responses and strategies then affect the individual’s cognitive processes, ultimately resulting in positive psychological changes through adaptation or assimilation. Post-traumatic growth has been demonstrated to manifest in a variety of ways, including the acquisition of new insights into life, an increase in personal power, the discovery of new possibilities, the establishment of intimate relationships with others, and spiritual transformation (17). Research has demonstrated that post-traumatic growth has the capacity to alleviate symptoms of post-traumatic stress, including anxiety and distress (18). Furthermore, it has been associated with an enhancement in positive psychological well-being and a reduction in psychological distress (19). Post-traumatic growth has been identified as a significant internal protective factor for mental health, with its level and rate of development proving to be a reliable predictor of mental health outcomes (20).

Many studies have found that individual differences can affect the level of post-traumatic growth (21, 22). However, current research on post-traumatic growth in patients with recent hysterectomy has not considered the differences between individuals undergoing the procedure. Examining heterogeneity among groups of patients can help to reflect differences in psychological development. Identifying subgroups with common symptoms can facilitate targeted screening and early detection, guide healthcare providers in developing personalised psychological interventions, and improve patients’ levels of post-traumatic growth, thereby enhancing their overall health (23).

A literature review revealed that post-traumatic growth is influenced by sociodemographic characteristics (e.g. age, occupation and educational attainment), disease-related factors (e.g. diagnosis type), external environmental factors and individual psychological factors (21, 24). Additionally, women who have undergone hysterectomies may experience stigmatisation, which involves feelings of shame and self-stigmatisation related to the procedure. This can lead to negative psychological experiences and poor social adaptation. Yang Yuan (25) found that young and middle-aged patients who underwent a hysterectomy experienced moderate levels of stigma. Previous studies have shown that high levels of stigma can result in cognitive changes, making patients more susceptible to negative thoughts such as self-denial (26). This exacerbates psychological distress and hinders post-traumatic growth, creating a vicious cycle (27, 28). According to the emotional-cognitive processing model of PTG, positive re-evaluation and other cognitive processes are key to achieving post-traumatic growth. However, stigma — defined as the negative self-evaluation experienced by patients — can affect cognitive processes, hinder adaptation to traumatic events and impact psychological growth. Therefore, stigma may hinder the post-traumatic growth of patients who have undergone a hysterectomy. However, the impact of stigma on the level of post-traumatic growth in patients with recent hysterectomy has not yet been explored in research.

In summary, this study aimed to understand the level of post-traumatic growth among patients with recent hysterectomy based on the emotional-cognitive processing model, and to use latent profile analysis to explore differences in post-traumatic growth levels among patients, as well as the relationship between stigma and these levels. The study findings will inform the development of measures to increase post-traumatic growth among patients with recent hysterectomy, thereby improving their mental health. The research hypotheses are as follows: (1) There is heterogeneity in post-traumatic growth levels among patients with recent hysterectomy. (2) Stigma predicts the levels of post-traumatic growth experienced by patients who have undergone hysterectomy, meaning patients with lower levels of stigma are more likely to be categorised as experiencing higher levels of post-traumatic growth.

## 2 Materials and methods

### 2.1 Design and sample

A cross-sectional survey was used in this study. The results were reported according to the Strengthening the Reporting of Observational Studies in Epidemiology (STROBE) guidelines. Convenience sampling was used to select patients who had undergone hysterectomy surgery in the gynaecology department of a tertiary hospital in Guangzhou, Guangdong Province, for the study. The inclusion criteria were: age of ≥18 years old (29); surgical procedure of total hysterectomy; clear consciousness, with certain reading, comprehension, and expression abilities or able to complete the questionnaire with the help of the investigator; informed consent, voluntary enrolment in the study, and signing of the informed consent form. The exclusion criteria included: currently participating in or having participated in a psychological intervention study within the past six months; those with comorbid critical illnesses(e.g. heart, lung, or renal failure) who are unable to fully cooperate; those with severe mental illnesses or psychiatric disorders or cognitive dysfunction resulting in an inability to communicate; and other major traumatic events in the family (e.g., bereavement, exposure to natural disasters, etc.).

The sample size was calculated according to the Kendall guidelines (30). In this study, there were 21 variables, and the sample size was calculated according to 5 times the total number of variables, taking into account a 10% loss-of-visit rate, resulting in a required sample size of at least 116 cases for this study. A total of 210 questionnaires were distributed for the final study. After excluding questionnaires with missing or problematic data, 202 valid questionnaires were recovered, with a valid recovery rate of 96.2%.

### 2.2 Instruments

#### 2.2.1 General demographic questionnaire

The research team designed it after a literature review, including age, marital status, childbearing status, education level, occupation, family financial income, place of residence, payment method, diagnosis of disease, time since diagnosis, type of surgery, and degree of knowledge of the disease.

#### 2.2.2 Post-traumatic growth inventory-Chinese version

The original scale was developed by Tedeschi et al. In this study, this study used the Chinese version of the scale translated and revised by Wang Ji et al. based on the accidental trauma population (31), including 5 dimensions of appreciation of life, personal strength, new possibilities, relatedness to others, and spiritual changes, with a total of 20 items. Each item was scored on a five-point Likert scale, and the higher the total score, the higher the level of post-traumatic growth. The Cronbach’s α of the revised dimensions and the total scale ranged from 0.611 to 0.874, with good reliability and validity, and has been widely used. The Cronbach’s α of the scale in this study was 0.937.

#### 2.2.3 Social impact scale

The Chinese version of the scale was revised by Pan et al (32). It consists of 24 items, including four dimensions: social rejection, social isolation, internalized shame, and financial insecurity. The scale is scored from “strongly disagree” to “strongly agree” in the order of 1 to 4, and the total score of the scale is 24 to 96, with higher scores indicating higher levels of stigma. The Cronbach’s α of the Chinese version of the scale was 0.85-0.90, and the Cronbach’s α of the scale in this study was 0.862.

### 2.3 Data collection and quality control

Data were collected on-site from February to September 2024 using a face-to-face approach. A uniformly trained researcher used a uniform instruction to inform patients of the method and precautions for filling out the questionnaire. For those who have difficulty in reading and writing, the researcher will help them to fill in the questionnaire by using the question-and-answer method. After the completion of the survey, the researcher checked the standardization and completeness of the filling on the spot and recovered the questionnaires after making sure that there were no errors. Patient disease-related information was filled in by the researcher based on the electronic medical record.

### 2.4 Data analysis

Latent profile analysis (LPA) is a type of latent class analysis which is primarily used to analyse continuous variables. It can use probability models to estimate and compare probabilities, determine various categories through fitting indicators and statistical tests, and is often used to identify latent groups with similar symptom experiences and characteristics (33, 34). Not only does it take into account the uncertainty of variables, it also classifies them more objectively, resulting in more accurate outcomes. It addresses the shortcomings of traditional ‘variable-centric’ statistical methods, which fail to clearly and explicitly observe the characteristics of individuals within different categories (35). This classification method provides a deeper understanding of the differences in post-traumatic growth among patients with recent hysterectomy, identifies the optimal classification for post-traumatic growth and highlights key populations. This helps healthcare professionals to develop more personalised intervention plans.

Mplus 8.3 and SPSS 25.0 were used to statistically analyse the data that were entered after double-checking. This study relied primarily on self-reporting by the research subjects, which may have introduced common method bias. Therefore, the Harman single-factor test was used to examine this bias in the scale items. SPSS 25.0 was used to conduct a descriptive analysis of the patients with recent hysterectomy’ demographic information, post-traumatic growth and stigma. The performance of normality tests is based on graphical methods, with histograms serving as the primary data representation. In the event of the histogram manifesting a bell-shaped curve, with a high peak in the middle and lower peaks at both ends, the data is considered to be approximately normally distributed. For normally distributed metric data, the mean ± standard deviation(M ± SD) was used, whereas the median and interquartile range were employed for non-normally distributed data. Categorical data were described using frequencies and percentages. Secondly, Mplus 8.3 was used to build a latent profile model with exogenous variables as scores on 20 entries of the post-traumatic growth inventory for patients with recent hysterectomy. The initial model comprised a single category, which was then expanded to encompass a progressively larger number of categories. This process was continued until the model fit indicators achieved optimal levels. The model fitting indicators include: ① information evaluation indexes included Aichaike information criterion (AIC), Bayesian information criterion (BIC) and sample corrected aBIC (adjusted aBIC, aBIC). The smaller the value, the better the model fit (36); ② classification indexes were Entropy, with the value range of 0 to 1, and the closer to 1 indicated that the classification was more accurate (37); ③likelihood ratio test indicator includes Lo-Mendell-Rubin Test (LMRT), and the Bootstrap Likelihood Ratio Test (BLRT). At *P* < 0.05, it means that k categories are better than the model with k-1 categories (38). The extant research indicates that BLRT is the optimal evaluation metric, followed by the BIC, the aBIC, and entropy (36, 39). This study will determine the optimal model based on the above-mentioned fitting metrics, combined with clinical practical significance and classification interpretability. In conclusion, the study utilised one-way analysis of variance (ANOVA) and chi-square tests to analyse continuous and categorical variables, respectively, with a view to comparing differences in sociodemographic characteristics between subgroups. Variables that demonstrated statistical significance in the one-way analysis were subsequently incorporated into a multiple logistic regression model. This model was utilised to identify the factors that influence the various categories of post-traumatic growth. Continuous variables were incorporated into the model as raw values, while categorical variables were systematically converted into dummy variables using SPSS 25.0. In this particular context, unordered categorical variables were designated as factor variables. The generation of dummy variables was undertaken automatically, with the maximum-coded category serving as the reference category. *P* < 0.05 indicated that the difference was statistically significant.

### 2.5 Ethical principles

Ethics approval was granted by the Ethics Committee of Guangdong Provincial Hospital of Traditional Chinese Medicine (YE2024 - 029) and was conducted in accordance with the Declaration of Helsinki. It was conducted with full informed sympathy from the participants, whose information could be guaranteed not to be disclosed and used only for research purposes. Participants may refuse or withdraw at any time and will not be charged or suffer any loss as a result.

## 3 Results

### 3.1 Common method bias test

The results of the Harman single-factor test demonstrated that 11 factor eigenvalues exceeded 1, with the maximum factor accounting for 27.416% of the variance, which is below the critical value of 40%. This finding suggests that there is an absence of significant common method bias in this study.

### 3.2 Participant characteristics

A total of 202 patients with recent hysterectomy, aged 33 to 77 years, with a mean age of (51.59 ± 8.43) years. Most of the patients were married (83.7%) and had previous childbearing experience (89.1%). 15.3% of the patients had an education level of elementary school and below, 25.2% of patients had a junior high school education, 29.2% had a high school or technical secondary school education, and 30.2% had a college education or above. Most patients’ families have a decent income, with 44.1% of patients’ families having a per capita monthly income of over 5,000 yuan (The USD/CNY rate is 7.1523). The majority of patients resided in urban areas (73.8%), and the vast majority of patients had medical insurance. Furthermore, 77.2% of patients had a basic understanding of their condition, and most patients underwent surgery for benign diseases (76.7%).

The normality of the data was established through the implementation of graphical methods. The histograms of the post-traumatic growth and stigma scale scores for patients who underwent hysterectomy demonstrated a bell-shaped curve, with elevated values at the midpoint and diminished values at both extremes. This finding indicates that the data are approximately normally distributed, thus necessitating the use of the mean ± standard deviation for graphical representation. The score for post-traumatic growth was 54.41 ± 17.98 points, and the score for stigma was 48.99 ± 6.79 points. Detailed information is given in **Table 1**.

**Table 1:** Demographic characteristics of the different profiles.

Variable		C1 (n=36)	C2 (n=108)	C3 (n=58)	*χ* ^2^/ *F*	*P*
Age	<40	0(0.0)	3(2.8)	3(5.2)	18.123^a^	<0.001
	40-59	23(63.9)	88(81.5)	53(91.4)		
	≥60	13(36.1)	17(15.7)	2(3.4)		
Marital status	Married	29(80.6)	88(81.5)	52(89.7)	2.154^a^	0.341
	Single/Divorce/Widowed	7(19.4)	20(18.5)	6(10.3)		
Childbearing status	Yes	4(11.1)	12(11.1)	6(10.3)	0.025^a^	0.988
	No	32(88.9)	96(88.9)	52(89.7)		
Education level	Elementary school and below	8(22.2)	19(17.6)	4(6.9)	13.252^a^	0.039
	junior high school	10(27.8)	29(26.9)	12(20.7)		
	High school or technical secondary school	12(33.3)	32(29.6)	15(25.9)		
	College degree or above	6(16.7)	28(25.9)	27(46.6)		
Occupation status	In-service/Employed	4(11.1)	31(28.7)	21(36.2)	7.920^a^	0.244
	Retired	16(44.4)	33(30.6)	16(27.6)		
	Unemployed or jobless	6(16.7)	16(14.8)	9(15.5)		
	Other	10(27.8)	28(25.9)	12(20.7)		
Average monthly household income (yuan)	<1000	4(11.1)	3(2.8)	2(3.4)	6.368^a^	0.370
	1000-2999	2(5.6)	12(11.1)	9(15.5)		
	3000-5000	13(36.1)	47(43.5)	21(36.2)		
	>5000	17(47.2)	46(42.6)	26(44.8)		
Place of residence	City	27(75.0)	86(79.6)	36(62.1)	12.299^a^	0.043
	County	3(8.3)	6(5.6)	10(17.2)		
	Township	2(5.6)	2(1.9)	6(10.3)		
	Rural	4(11.1)	14(13.0)	6(10.3)		
Payment methods	At own expense	3(8.3)	2(1.9)	2(3.4)	5.313^a^	0.491
	Rural cooperative medical care	3(8.3)	6(5.6)	5(8.6)		
	Resident medical insurance	13(36.1)	34(31.5)	17(29.3)		
	Employee medical insurance	17(47.2)	66(61.1)	34(58.6)		
Degree of knowledge of the disease	Basic understanding	25(69.4)	85(78.7)	46(79.3)	1.517^a^	0.468
	Partial understanding	11(30.6)	23(21.3)	12(20.7)		
Disease diagnosis	Benign	24(66.7)	85(78.7)	46(79.3)	2.494^a^	0.287
	Malignant	12(33.3)	23(21.3)	12(20.7)		
Type of surgery	Laparoscopic surgery	25(69.4)	100(92.6)	53(91.4)	11.925^a^	0.002
	Non-laparoscopic surgery	11(30.6)	8(7.4)	5(8.6)		
Other chronic diseases	Yes	25(69.4)	78(72.2)	43(74.1)	0.245^a^	0.885
	No	11(30.6)	30(27.8)	15(25.9)		
Time since diagnosis	<1 year	17(47.2)	40(37.0)	25(43.1)	3.857^a^	0.426
	1-5 years	7(19.4)	39(36.1)	19(32.8)		
	>5years	12(33.3)	29(26.9)	14(24.1)		
SIS		53.36±4.01	49.39±5.22	45.53±8.77	17.696^b^	<0.001
Social rejection		18.22±1.87	17.06±2.31	15.74±3.83	9.335^b^	<0.001
Social isolation		15.81±1.86	14.36±2.13	12.67±3.13	19.610^b^	<0.001
Internalized shame		12.56±1.48	11.65±1.76	10.48±2.30	14.307^b^	<0.001
Financial insecurity		6.78±1.57	6.31±1.07	6.64±1.37	2.375^b^	0.096

a : *χ* ^2^, : *H*; C1, negative growth group; C2, low-transformation-moderate growth group; C3, positive growth group; SIS, Social Impact Scale.

### 3.3 Results of latent profile analysis

**Figure 1:**
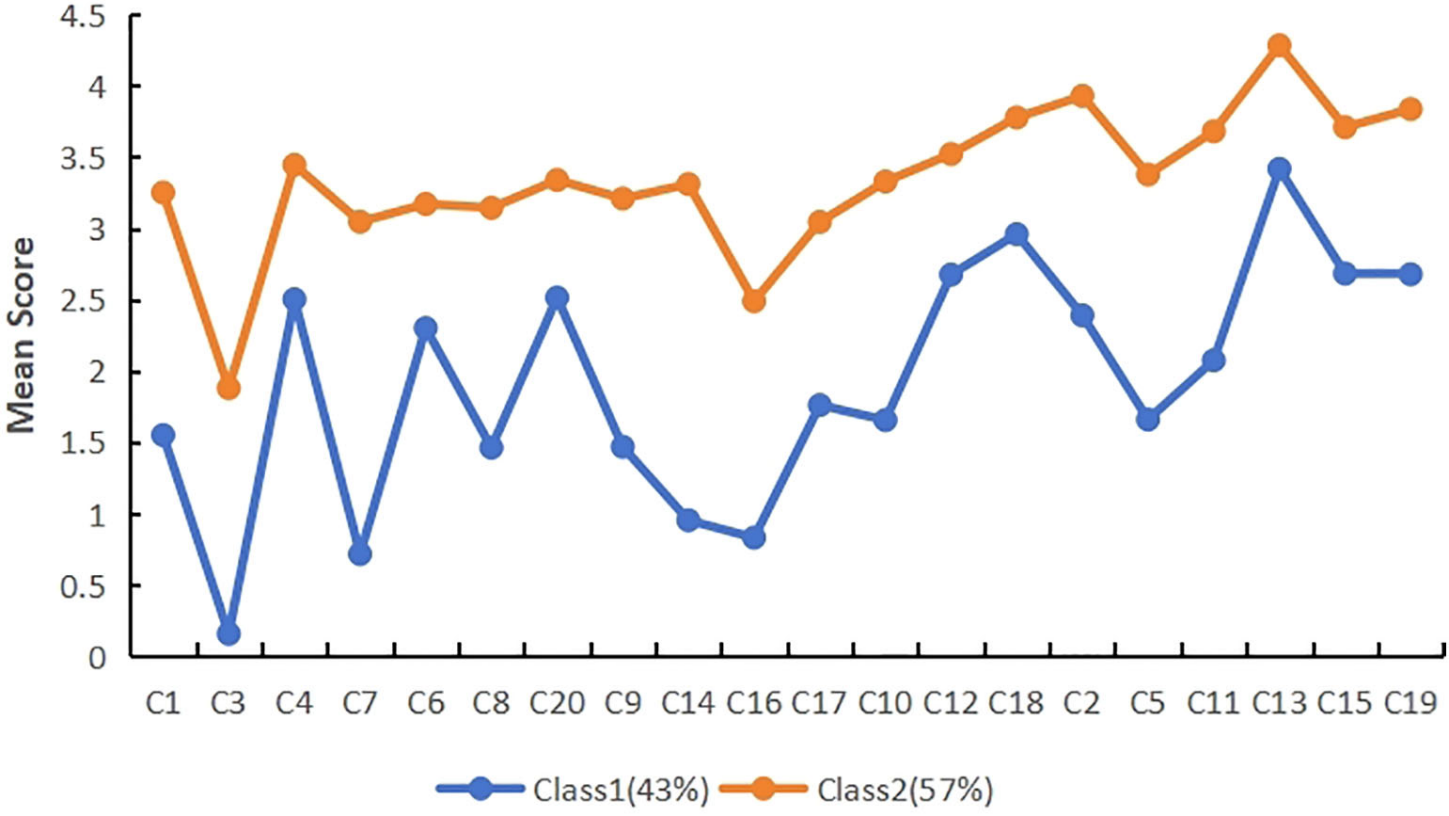
Two-profile modeling of post-traumatic growth.

**Figure 2:**
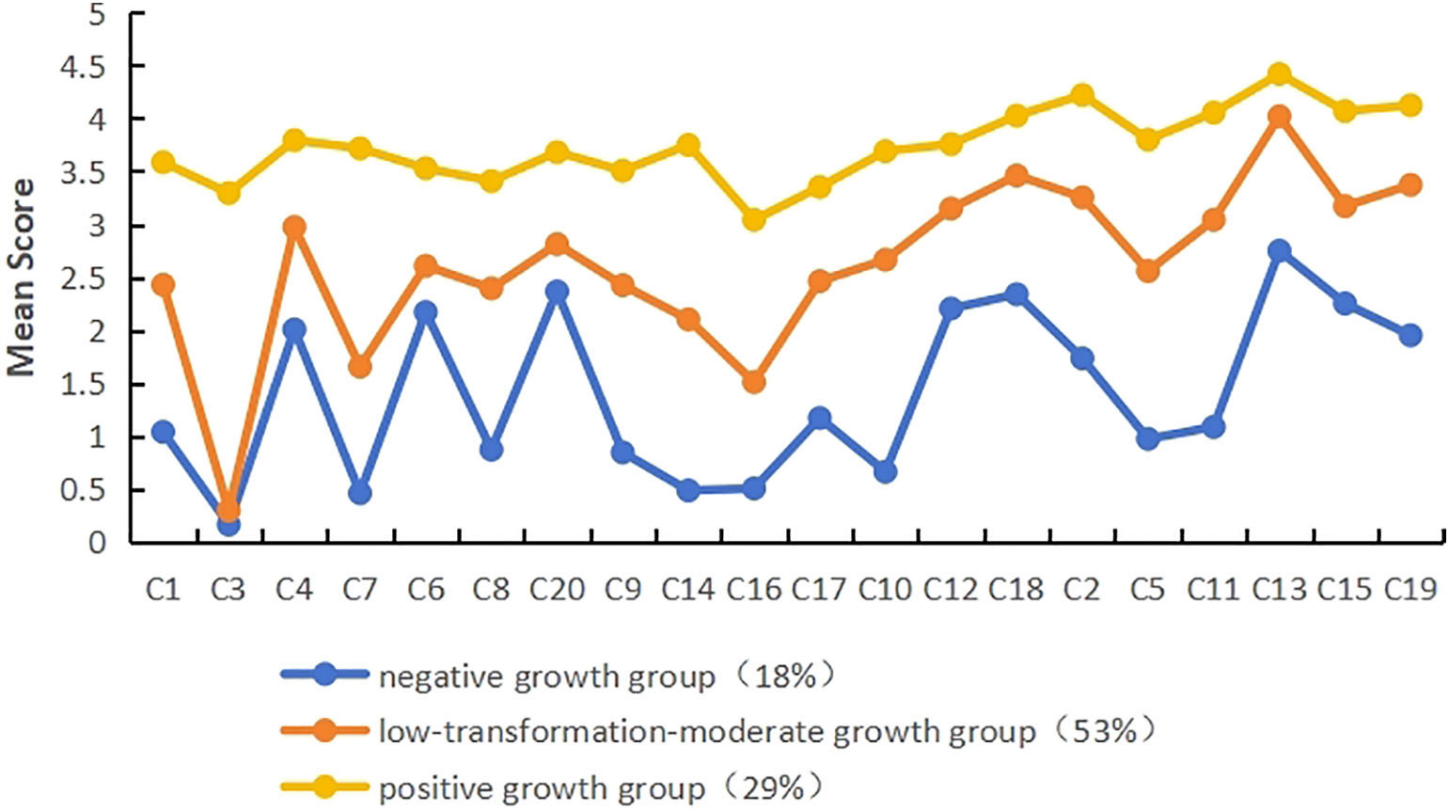
Three-profile modeling of post-traumatic growth (Note:spiritual changes: c1,c3,c4,c7; relatedness to others: c6,c8,c20; new possibilities: c9,c14,c16,c17; personal strength: c10,c12,c18; appreciation of life: c2,c5,c11,c13,c15,c19).

Latent profile analysis was carried out using the mean scores of the 20 items of post-traumatic growth as exogenous variables, and a total of four models were fitted, and the fitting indexes of each model are shown in **Table 2**. The results showed that as the number of profile increased, the values of AIC, BIC, and aBIC gradually decreased. The BLRT test values of all four models reached significance (*P* < 0.001), indicating that the larger the number of latent profile, the better the model fit. The entropy values all exceed 0.8, with the 3-profile model having the highest entropy value, indicating the highest classification accuracy and relatively better posterior probability. Subsequent analysis indicated that, in comparison with the 2-profile model, the 3-profile model exhibited a superior capacity to differentiate the subcategories of post-traumatic growth in patients with recent hysterectomy. The differences in post-traumatic growth scores among the three categories were more significant and clinically meaningful, as demonstrated in **Figures 1**, **2**. The mean membership probability of Model 3 was found to be between 98.1% and 99.2%, thus indicating reliable fitting results. Furthermore, **Supplementary Figure S1** presents the standard plot of fitting information. As one moves from the 3-profile onwards, it becomes evident that the enhancement of each fitting index reaches a point of considerable stabilisation. Consequently, on the basis of the fitting indices and the practical significance of classification, model 3 is selected as the optimal model (23).

**Table 2:** Classification of potential fitting models.

Profile	AIC	BIC	aBIC	*P*	Entropy	Proportion
				LMRT BLRT		
1	13663.084	13795.414	13668.686	—	—	1.00
2	12545.739	12747.543	12554.283	0.042 <0.001	0.928	0.43/0.57
3	12059.898	12331.176	12071.383	0.136 <0.001	0.966	0.18/0.53/0.29
4	11826.577	12167.328	11841.003	0.098 <0.001	0.948	0.10/ 0.41/0.32/0.17

### 3.4 Characterization and naming of latent profile

As shown in **Figure 2**, the distribution of scores on the 20 items for the three profiles of post-traumatic growth in patients with recent hysterectomy showed different characteristics. The profiles were named according to their score characteristics. Patients in class 1 (C1) had significantly lower scores on each of the items than the other classes and were named the “negative growth group”, with a total of 36 (18%); patients in class 2 (C2) were in the middle of the classes with moderate scores in each dimension, with the lowest score in the spiritual changes dimension, so they were named the “low-transformation-moderate growth group”, with a total of 108 (53%); patients in class 3 (C3) had the highest scores in each dimension, and the overall trend was relatively stable, so they were named the “positive growth group”, with a total of 58 (29%).

To test the validity of the results of the latent profile analysis, a one-way ANOVA was conducted with the different profiles of post-traumatic growth as the independent variable. The results showed that the difference in post-traumatic growth scores across latent profiles was statistically significant (*P* < 0.05), as shown in **Table 3**.

**Table 3:** Differences in post-traumatic growth scores across profiles.

Items	C1	C2	C3	*F*	*P*
Post-traumatic Growth	27.92 ± 9.92	52.28 ± 7.66	74.83 ± 9.64	330.933	<0.001
personal strength	5.19 ± 2.44	9.27 ± 1.79	11.47 ± 1.92	114.448	<0.001
new possibilities	2.97 ± 2.46	8.47 ± 3.14	13.67 ± 3.36	135.621	<0.001
relatedness to others	5.39 ± 2.43	7.80 ± 2.29	10.64 ± 2.84	52.341	<0.001
spiritual changes	3.64 ± 2.68	7.34 ± 2.67	14.38 ± 2.46	219.492	<0.001
appreciation of life	10.72 ± 4.22	19.40 ± 2.70	24.67 ± 2.97	225.928	<0.001

C1, negative growth group; C2, low-transformation-moderate growth group; C3, positive growth group.

### 3.5 The predictive role of general information and stigma on latent profiles of post-traumatic growth

The results of one-way ANOVA showed statistically significant (*P* < 0.05) differences in scores of post-traumatic growth of patients with recent hysterectomy with different profiles in terms of age, level of education, place of residence, and type of surgery. In addition, substantial disparities were observed among the potential categories of post-traumatic growth with respect to total stigma scores (*F*=17.696, *P*<0.001), social rejection (*F*=9.335, *P*<0.001), social isolation (*F*=19.610, *P*<0.001), and internalized shame (*F*=14.307, *P*<0.001). Furthermore, significant variations were identified among the latent categories of post-traumatic growth. Patients in the negative growth group demonstrated the highest total scores on the scale for stigma, social rejection, social isolation, and internalized shame, as shown in **Table 1**.

Multivariate logistic regression analyses were conducted using the statistically significant differences in the one-way ANOVA of variance as the independent variables, and the three profiles of post-traumatic growth as the dependent variables, employing the “positive growth group” as a reference (In the event of multi-class independent variables being present during the execution of a logistic regression analysis, the SPSS software will, by default, refer to the maximum-coded classification variable). The independent variables are assigned values: 1.age: <40 = 1, 40-59 = 2, ≥60 = 3; 2.level of education: elementary school and below = 1, junior high school = 2, high school or technical secondary school = 3, college degree or above = 4; 3.places of residence: city = 1, county = 2, township = 3, rural = 4; 4.type of surgery: laparoscopic surgery = 0, non-laparoscopic surgery = 1; 5.Social rejection, social isolation, and internalized shame are brought in at their original value.

The results demonstrated that, in comparison with the positive growth group, patients aged 40–59 years who underwent hysterectomy were more likely to be classified into the negative growth group (*OR* = 0.164, *P* = 0.042). Furthermore, patients with high scores on the internalized shame dimension (*OR*=1.539, *P*=0.015) and social isolation dimension (*OR*=1.323, *P*=0.044) were more likely to belong to the negative growth group. In contrast to the positive growth group, educational attainment at the elementary school level or below (*OR* = 4.196, *P* = 0.032) and junior high school level (*OR* = 2.766, *P* = 0.046) were predictive factors for the low transformation-moderate growth group, as shown in **Supplementary Table S1**.

## 4 Discussion

The accurate assessment and intervention in the postoperative psychological state of patients with recent hysterectomy is of great significance for maintaining their overall health. The findings of this study indicate that the post-traumatic growth scores of patients with recent hysterectomy are below average (40). This suggests that there is potential for enhancement in the level of post-traumatic growth among this demographic. The present study utilised the LPA to identify three distinct categories of post-traumatic growth among patients with recent hysterectomy, thereby underscoring the heterogeneity in post-traumatic growth. Among the patients with recent hysterectomy, 18% were found to be part of the negative growth group. Patients in this group exhibited the lowest post-traumatic growth scores, encountered difficulties in engaging in positive self-regulation, demonstrated a heightened propensity for psychological distress, and exhibited a reluctance to actively express themselves or seek support (41). The low transformation-moderate growth group accounted for 53%, representing the largest proportion and thus serving as the primary target for intervention. This group of patients exhibited growth, albeit with minimal cognitive changes, which made it challenging for them to identify new possibilities and initiate a new life. It is recommended that healthcare providers offer targeted guidance to assist patients in cultivating new interests and formulating new life plans. Furthermore, it is imperative to monitor their psychological dynamics in order to avert a reversion to the negative growth group. The positive growth group (29%) demonstrated higher scores, with patients exhibiting resolute beliefs and clearly defined goals. Patients in this category have been observed to demonstrate a stronger propensity for active coping mechanisms and a more pronounced capacity for adaptation in the face of adversity (42). Healthcare providers can encourage patients in the positive growth group to interact and share experiences with patients in the other two groups, thus fostering peer support and enhancing others’ confidence in recovery while maintaining their own high level of growth. In clinical practice, healthcare providers have the capacity to assess patients’ psychological states based on the characteristics of different categories of post-traumatic growth. This ability enables the development of more practical and tailored care plans, and the provision of precise care.

The present study found that, in comparison with the negative growth group, patients aged 40–59 who underwent uterine resection were more likely to demonstrate positive growth. This may be because older patients have poor recovery abilities and cannot respond to their feelings and needs in a timely manner. Consequently, this may have an adverse impact on their psychological well-being (43). This also suggests that younger patients are better able to accept new things than older patients (44). They are more proactive in seeking methods and avenues to promote self-transformation, thereby making more significant progress in their psychological growth. Therefore, healthcare providers should actively inquire about the needs of older patients during treatment, help them to understand their conditions and boost their confidence in recovery. Regular follow-ups should also be conducted after discharge to ensure continuity of care. At the same time, encourage family members to support patients actively and encourage caregivers to participate in formulating treatment plans, in order to promote patients’ physical and psychological adjustment (45). Additionally, patients with recent hysterectomy who had an educational attainment level of elementary school or below, or junior high school, were more likely to be classified into the low transformation-moderate growth group than those in the positive growth group. Those with higher levels of education typically possess stronger information processing capabilities and have access to a wider range of avenues and methods for obtaining social support. Consequently, they are better able to utilise resources for regulation, effectively buffering the harm caused by traumatic events, and are thus more likely to experience positive growth (46). Healthcare providers should assess the level of healthcare-related support required by patients with lower levels of education. They should also be more patient with these patients and provide personalised disease education to help alleviate their psychological distress.

Furthermore, the study results indicate that stigma is a significant predictor of potential categories of post-traumatic growth among patients with recent hysterectomy. Patients who exhibit elevated scores on the internalized shame and social isolation dimensions are more likely to be classified as belonging to the negative growth group. Analysing the reasons, stigma can affect an individual’s communication and interaction with others, limiting their ability to seek help during adversity (27, 47). This is detrimental to positive changes in their cognitive behaviour. High scores on the internalized shame and social isolation dimensions indicate that patients with recent hysterectomy harbour negative stereotypes about themselves, making it difficult for them to reconnect with society (25). Healthcare professionals should implement targeted interventions based on patients’ varying levels of knowledge and coping abilities. Patients with lower levels of education may develop negative self-perceptions due to limitations in their knowledge, thereby exacerbating their sense of stigma associated with their condition. To help such patients, hospitals should strengthen public education on diseases and surgery to enhance understanding among patients and society, thereby helping patients gain greater respect and support. Patient exchange meetings and themed lectures can be held to answer patients’ questions and clarify their doubts. Post-discharge follow-up should also be conducted to provide individualised discharge guidance, such as setting up WeChat groups. Encouraging family members to participate can promote mutual understanding, which is beneficial for reducing stigma associated with illness. Secondly, we should actively guide key patients with poor problem-solving and adaptability skills to view their illness from multiple perspectives and adjust their psychological state. We should help patients to establish a good social support system and encourage their family members to provide adequate support. Family members should also participate in the patient’s treatment and care, as this can improve communication, help patients rebuild their confidence and achieve psychological growth, and reduce their stigma.

The findings of this study indicate that screening based on post-traumatic growth can be utilised in clinical practice to identify patients at risk of mental health problems and provide tiered intervention measures. For instance, the provision of enhanced support to patients in the negative growth group and the low transformation-moderate growth group is intended to encourage positive cognitive evaluation and effective coping mechanisms among patients. Furthermore, it is imperative to fortify the care provided to patients within the positive growth group, with the objective of averting a diminution in their post-traumatic growth levels.

### 4.1 Limitations and future research

However, there were some limitations in this study: due to limitations in terms of time, human resources and material conditions, the study only collected samples from one hospital, which restricts its representativeness. Further validation of the results through larger-scale studies with a broader sample size is required to strengthen the persuasiveness and generalisability of the findings. Secondly, the study relied on patient self-reports, which may have introduced bias into the results. To validate the results in future, objective indicators such as biological markers should be included. At the same time, this study is only a cross-sectional survey, so it cannot demonstrate causal relationships or changing trends between variables. Furthermore, many factors affect the post-traumatic growth of patients with recent hysterectomy, and this study may not have considered all of these factors. Future studies are recommended to include more influencing factors and collect data from multiple time points through prospective longitudinal studies, in order to observe changing trends in patients’ post-traumatic growth. Additionally, analysis methods such as structural equation modelling could be employed to explore the interaction paths between variables in depth. In addition, high-quality randomised controlled trials should be conducted to validate the effectiveness of personalised intervention measures. This would provide a comprehensive understanding of the psychological changes experienced by patients with recent hysterectomy. It would allow for the more effective management of their psychological issues and provide a theoretical basis for developing non-medical psychological health intervention models for these patients.

## 5 Conclusion

Research has found that post-traumatic growth in patients with recent hysterectomy can be categorised into three groups: negative growth group; low transformation–moderate growth group; and positive growth group. The largest proportion is accounted for by the low transformation–moderate growth group. Younger patients with higher levels of education and lower scores on the SIS tend to experience better post-traumatic growth after hysterectomy. Future research should prioritise developing tools to screen patients’ psychological states based on post-traumatic growth, as well as conducting randomised controlled trials to assess the effectiveness of personalised intervention measures, such as structured psychological education interventions. Such measures could help patients to identify and resolve psychological issues, improve their level of post-traumatic growth and enhance their quality of life.

## Data Availability

The data analysed in this study is subject to the following licenses/restrictions: The original contributions presented in the study are included in the article/**Supplementary Material**. Further inquiries can be directed to the corresponding authors. Requests to access these datasets should be directed to Xiaojie Zheng, 5888880@qq.com or Dingrong Qiu, gzqdrong@126.com.
