# Correction: Latent profiles of post-traumatic growth in patients with recent hysterectomy: psychosocial predictors and stigma-associated outcomes

**DOI:** 10.3389/fpsyt.2025.1682361

**Published:** 2025-09-26

**Authors:** Yuqing Sun, Shirong Wu, Qunfeng Zou, Xiuzhen Fu, Silu Liu, Gongna Chen, Xiaojie Zheng, Dingrong Qiu

**Affiliations:** ^1^ The Second Clinical School of Medicine, Guangzhou University of Chinese Medicine, Guangzhou, China; ^2^ Department of Nursing, The Second Affiliated Hospital of Guangzhou University of Chinese Medicine, Guangzhou, China; ^3^ Department of Sports Medicine, The Second Affiliated Hospital of Guangzhou University of Chinese Medicine(Er Sha Island), Guangzhou, China

**Keywords:** hysterectomy, latent class analysis, patients, post traumatic growth, psychological, social stigma

The order of authors in the author list of the published paper was erroneously given as: “Yuqing Sun1, Shirong Wu1, Qunfeng Zou1, Dingrong Qiu2, Xiuzhen Fu2, Silu Liu3, Gongna Chen3 and Xiaojie Zheng3*”

The correct author list reads:

“Yuqing Sun^1^, Shirong Wu^1^, Qunfeng Zou^1^, Xiuzhen Fu^2^, Silu Liu^3^, Gongna Chen^3^, Xiaojie Zheng^3*^ and Dingrong Qiu^2*^”

The **Author Contributions** statement has been corrected to read:

“YS: Conceptualization, Data curation, Formal Analysis, Software, Writing – original draft, Writing – review & editing. SW: Conceptualization, Data curation, Formal Analysis, Software, Writing – original draft, Writing – review & editing. QZ: Conceptualization, Data curation, Formal Analysis, Supervision, Validation, Writing – review & editing. XF: Funding acquisition, Methodology, Project administration, Resources, Writing – review & editing. SL: Conceptualization, Data curation, Supervision, Validation, Writing – review & editing. GC: Conceptualization, Data curation, Supervision, Validation, Writing – review & editing. XZ: Methodology, Project administration, Resources, Writing – review & editing. DQ: Funding acquisition, Methodology, Project administration, Resources, Writing – review & editing.”

The **Citation** has been updated to read:

“Sun Y, Wu S, Zou Q, Fu X, Liu S, Chen G, Zheng X and Qiu D (2025) Latent profiles of post-traumatic growth in patients with recent hysterectomy: psychosocial predictors and stigma-associated outcomes. *Front. Psychiatry* 16:1552946. doi: 10.3389/fpsyt.2025.1552946”

The **Copyright** statement has been updated to read:

“© 2025 Sun, Wu, Zou, Fu, Liu, Chen, Zheng and Qiu. This is an open-access article distributed under the terms of the Creative Commons Attribution License (CC BY). The use, distribution or reproduction in other forums is permitted, provided the original author(s) and the copyright owner(s) are credited and that the original publication in this journal is cited, in accordance with accepted academic practice. No use, distribution or reproduction is permitted which does not comply with these terms.”

Author “Xiaojie Zheng” was erroneously assigned as sole corresponding author. The correct corresponding authors are “Xiaojie Zheng” and “Dingrong Qiu”. The **Correspondence** section has been updated to read:

“Xiaojie Zheng, 5888880@qq.com; Dingrong Qiu, gzqdrong@126.com“

The **Data availability statement** has been updated to read:

“The data analysed in this study is subject to the following licenses/restrictions: The original contributions presented in the study are included in the article/Supplementary Material. Further inquiries can be directed to the corresponding authors. Requests to access these datasets should be directed to Xiaojie Zheng, 5888880@qq.com or Dingrong Qiu, gzqdrong@126.com.”

The original version of this article has been updated.

