# Supplementary material for: Latent profiles of post-traumatic growth in patients with recent hysterectomy: psychosocial predictors and stigma-associated outcomes
Source: Front Psychiatry. 2025 Jul 28;16:1552946. doi: 10.3389/fpsyt.2025.1552946 (PMC12336223; doi:10.3389/fpsyt.2025.1552946)

Table S1 The results of multiple logistic regression of profiles

| Items | | negative growth group | | | | | | low-transformation-moderate growth group | | | | | |
| --- | --- | --- | --- | --- | --- | --- | --- | --- | --- | --- | --- | --- | --- |
|  |  | β | *SE* | Wald χ^2^ | *P* | *OR* | *95%CI* | β | *SE* | Wald χ^2^ | *P* | *OR* | *95%CI* |
| Age | 40-59 | -1.806 | 0.888 | 4.137 | 0.042 | 0.164 | （0.029~0.936） | -1.019 | 0.808 | 1.592 | 0.207 | 0.361 | （0.074~1.758） |
|  | ≥60 |  |  |  |  |  |  |  |  |  |  |  |  |
| Education level | Elementary school and below | 1.685 | 0.882 | 3.650 | 0.056 | 5.390 | （0.957~30.354） | 1.434 | 0.667 | 4.624 | 0.032 | 4.196 | （1.135~15.505） |
|  | Junior high school | 1.251 | 0.756 | 2.736 | 0.098 | 3.495 | （0.794~15.391） | 1.018 | 0.511 | 3.967 | 0.046 | 2.766 | （1.016~7.530） |
|  | College degree or above |  |  |  |  |  |  |  |  |  |  |  |  |
| Internalized shame |  | 0.431 | 0.177 | 5.941 | 0.015 | 1.539 | （1.088~2.178） | 0.110 | 0.115 | 0.923 | 0.337 | 1.116 | （0.892~1.397） |
| Social isolation |  | 0.280 | 0.139 | 4.055 | 0.044 | 1.323 | （1.007~1.738） | 0.161 | 0.098 | 2.703 | 0.100 | 1.175 | （0.969~1.424） |

SIS: Social Impact Scale; Use the positive growth group as a reference.

Figure S1 Four Model Parameter Variations


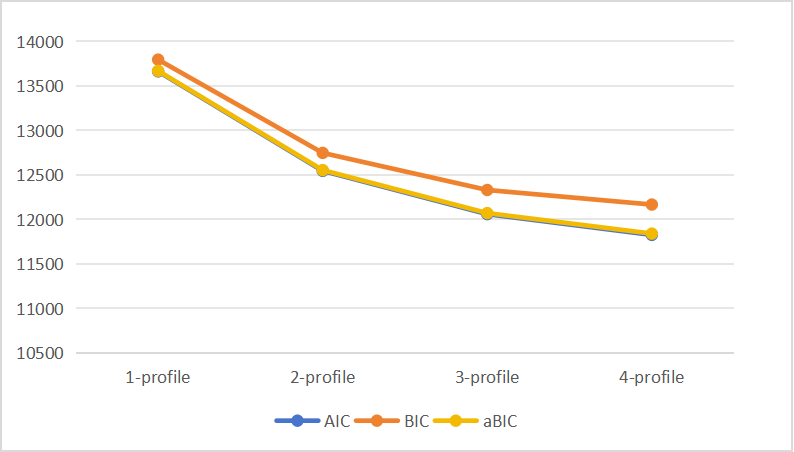

Supplement: Supplementary file 1 [file SupplementaryFile1.docx]
